# Evaluation of lateral and anterior center-edge angles according to sex and anterior pelvic plane tilt angle: a three-dimensional quantitative analysis

**DOI:** 10.1186/s13018-023-03763-z

**Published:** 2023-04-05

**Authors:** Kee-Bum Hong, Woo-suk Lee, Kyutae Kang, Kyoung Tak Kang, Byung Woo Cho

**Affiliations:** 1 Department of Orthopaedic Surgery, Gangnam Severance Hospital, Yonsei University College of Medicine, Seoul, Republic of Korea; 2 Department of Mechanical Engineering, Yonsei University, 50 Yonsei-ro, Seodaemun-gu, Seoul, Republic of Korea

**Keywords:** Anterior center-edge angle, Lateral center-edge angle, Acetabular coverage, Anterior pelvic plane tilt angle, Sex differences

## Abstract

**Background:**

This study aimed to quantitatively evaluate lateral center-edge angle (LCEA) and anterior center-edge angle (ACEA) according to sex and the anterior pelvic plane (APP) tilt angle and analyze the correlation between these measurements and acetabular coverage.

**Methods:**

Computed tomography scans of 71 adults (38 men and 33 women) with normal hip joints were obtained. LCEA, anterior ACEA, and acetabular coverage were measured with APP tilt every 5° from − 30° to + 30° and were compared between the sexes. The correlation between acetabular coverage and LCEA/ACEA was also analyzed.

**Results:**

(1) LCEA, ACEA, and acetabular coverage were statistically larger in men than in women at all APP tilt angles (with the exception of acetabular coverage ≥ 25°). (2) LCEA, ACEA, and acetabular coverage differed according to APP tilt angle. LCEA and acetabular coverage showed maximum values at 10°. ACEA showed a tendency to increase by an average of 3.6° for every 5° increase in the APP tilt angle. LCEA demonstrated strong and very strong associations across all APP tilting angles, whereas ACEA showed a moderate association at angles ≥ 15° in men and ≥ 30° in women.

**Conclusions:**

The LCEA and ACEA are adequate measurement methods that reflect actual acetabular coverage unless the pelvis is tilted excessively anteriorly. While pelvic tilting does not need to be considered for LCEA within the physiologic range, it should always be taken into account for ACEA, as it increases by an average of 3.6° for every 5° increase in APP tilt angle.

**Level of evidence:**

Level III: retrospective cohort study.

## Background

The degree of acetabular coverage of the femoral head is associated with the occurrence of hip joint diseases. In cases of undercoverage such as hip dysplasia, the reduced weight bearing surface increases the contact stress, leading to damage to the labrum and articular cartilage [15]. Conversely, in the case of overcoverage, the labrum is crushed between the acetabular rim and femoral neck due to femoroacetabular impingement causing tears and degeneration [2]. Therefore, several methods have been proposed to evaluate acetabular coverage.

The lateral center-edge angle of Wiberg (LCEA) [30] and Lequesne's acetabular index (anterior center-edge angle or ACEA) [14] are commonly used methods to assess the degree of acetabular coverage. Angles less than 20° are considered undercoverage [6], and those greater than 39° are considered overcoverage [27, 28]. However, information provided by LCEA and ACEA is limited for the following reasons. First, simple radiographs in which LCEA and ACEA are measured are two-dimensional (2D) images and therefore cannot precisely reflect the three-dimensional (3D) coverage of the acetabulum [13, 33]. Therefore, it is impossible to evaluate structures other than those shown on anteroposterior radiographs and false profile views. Second, LCEA and ACEA do not reflect pelvic tilting. When pelvic tilting changes due to factors such as spinal disease or posture, the acetabulum rotates accordingly, and the measurements on the 2D X-ray image change. Therefore, if acetabular coverage were evaluated using the standard value without taking these factors into account, the evaluation would be incorrect.

The impact of pelvic orientation on LCEA and ACEA has been reported in previous cadaveric studies [8, 20, 26]. However, the results of these studies regarding LCEA are inconsistent in the previous literature, possibly due to a lack of control for sex and ethnicity [3, 18], as well as due to differences in the definition of the neutral position. Therefore, the purpose of this study is to (1) quantitatively evaluate the LCEA and ACEA according to the anterior pelvic plane (APP) tilt angle and compare the results between sexes, and (2) analyze the correlation between LCEA/ACEA and actual acetabular coverage. We hypothesize that acetabular coverage and LCEA/ACEA would differ according to APP tilt angle and sex.

## Methods

### Subject recruitment

We conducted a retrospective study using full-length lower extremity computed tomography scan images of Korean adults aged 20–50 years who were diagnosed with lower extremity vascular disease at our institution between June 2020 and May 2021. The exclusion criteria included patients with: (1) hip joints with osteoarthritis or osteophytes, (2) hip joints with previous surgery, dysplasia, congenital anomalies, or traumatic deformities, and (3) pelvis and femurs that were not included in imaging. Finally, 142 hips from 71 participants (38 men and 33 women) were enrolled in the study.

### 3D model reconstruction

3D model reconstruction and measurements were performed by using Mimics and 3-Matic software (Materialize, Leuven, Belgium). Spatial orientation was as follows:

- *X*-axis: the line between both femoral head centers
- *Z*-axis: the line perpendicular to the *X*-axis and included in APP
- *Y*-axis: the line perpendicular to the *X*- and *Z*-axes

The default posture was defined as that in which the femur's mechanical axes were parallel to the *Z*-axis and perpendicular to the *X*-axis.

### Acetabular measurements in 3D models

**Fig. 1:**
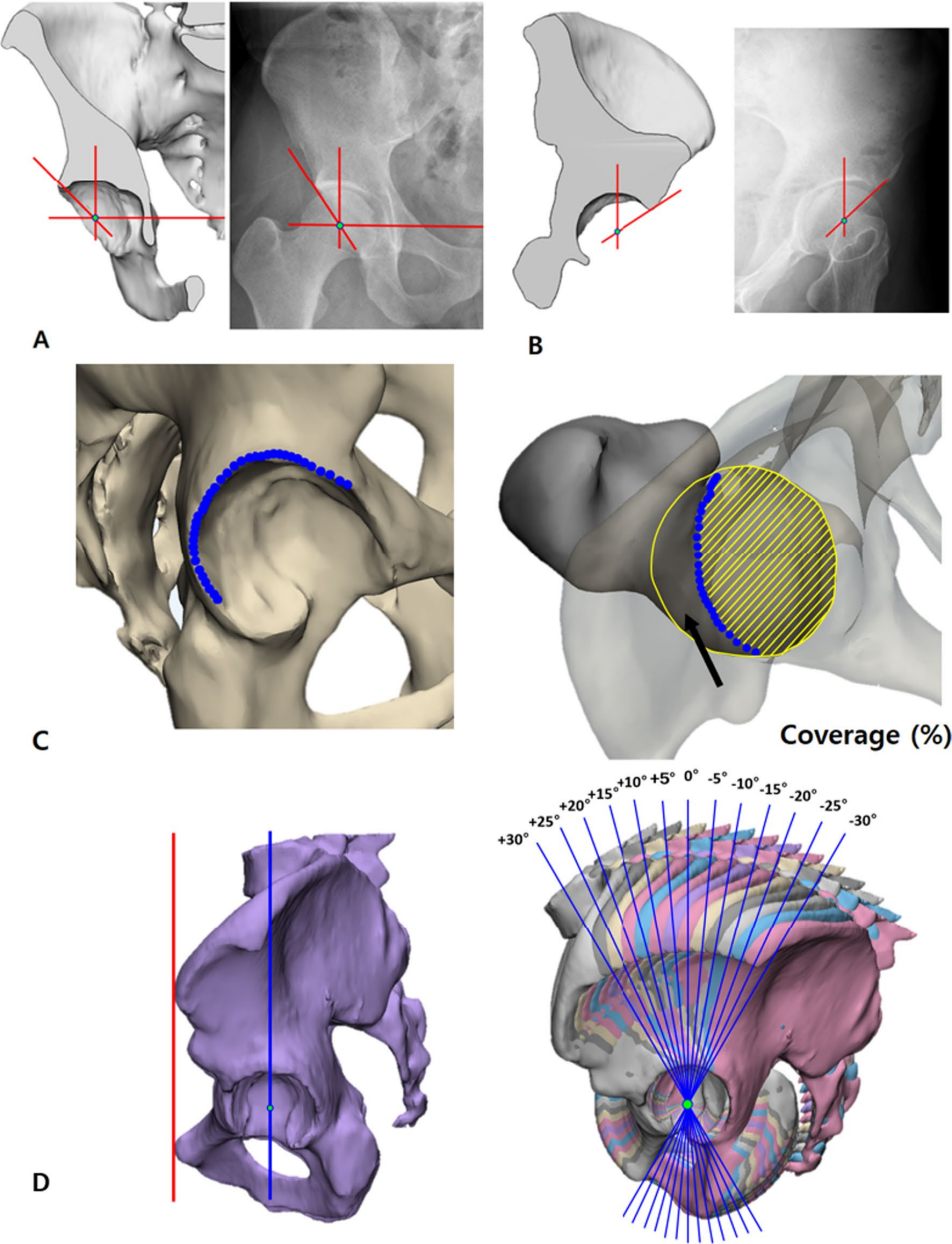
**A**–**D** The figures show the measurements of **A** lateral center-edge angle (LCEA), **B** anterior center-edge angle (ACEA), **C** acetabular coverage, and **D** anterior pelvic plane (APP) tilting from − 30° to + 30°

1. LCEA (sourcil LCEA).
  The pelvic model was cut into a plane that was parallel to the APP, containing the most laterally protruding point on the acetabular rim. On this plane, the angle between the line perpendicular to the *X*-axis and the line from the center of the femoral head to the edge of the sclerotic sourcil was measured [31] (Fig. 1A).
2. ACEA.
  The pelvic model was cut into a plane that was parallel to the 65°-rotated APP and contained the most anterolaterally protruding point on the acetabular rim. On this plane, the angle between the line perpendicular to the *XY* plane and the line from the center of the femoral head to the anterolateral edge of the acetabular rim was measured (Fig. 1B).
3. Acetabular coverage.
  The acetabular coverage area of the femoral head in the horizontal plane was calculated with the boundary of the femoral head which was defined based on the superior hemisphere. Coverage was defined as the area covered by the acetabulum/area of the femoral head (Fig. 1C).

LCEA, ACEA, and acetabular coverage were measured every 5° from − 30° to + 30° of APP tilt angles bilaterally (Fig. 1D). Positive values indicate forward tilting, while negative values represent backward tilting. Physiologic APP tilt angle range was set from − 10° to + 5°.

### Statistical analyses

An independent two-sample *t*-test was used to compare the differences in CEAs and acetabular coverage between sexes. Pearson’s correlation analysis was used to analyze the relationship between acetabular coverage and CEAs. The intra- and inter-observer reliabilities of the measurements were assessed using intraclass correlation coefficients. Statistical analyses were performed using SPSS (version 25.0, IBM Inc., Armonk, NY, USA), and statistical significance was set at *p* < 0.05.

## Results

The LCEA and ACEA were significantly larger in men than in women at all APP tilt angles. Acetabular coverage was also significantly larger in men than in women at all APP tilt angles, except at + 25° and + 30° (Table 1).

**Table 1:** Comparison of acetabular measurements between sexes according to APP tilt angle

APP tilt angle (°)	Variables	Overall	Men (*n* = 76)	Women (*n* = 66)	*p* value
− 30	ACEA	9.61 ± 8.76	11.71 ± 9.50	7.19 ± 7.17	**0.002**
	LCEA	26.95 ± 7.67	30.22 ± 7.26	23.18 ± 6.32	**< 0.001**
	Coverage	0.65 ± 0.09	0.67 ± 0.09	0.63 ± 0.08	**0.006**
− 25	ACEA	13.62 ± 8.83	15.86 ± 9.49	11.06 ± 7.25	**0.001**
	LCEA	28.61 ± 7.38	31.67 ± 7.01	25.09 ± 6.15	**< 0.001**
	Coverage	0.69 ± 0.09	0.71 ± 0.09	0.67 ± 0.08	**0.021**
− 20	ACEA	17.53 ± 9.02	19.95 ± 9.54	14.75 ± 7.53	**0.001**
	LCEA	30.27 ± 7.19	33.11 ± 6.97	27.00 ± 5.97	**< 0.001**
	Coverage	0.73 ± 0.10	0.75 ± 0.09	0.72 ± 0.09	**0.041**
− 15	ACEA	21.70 ± 9.10	24.16 ± 9.70	18.87 ± 7.48	**< 0.001**
	LCEA	31.66 ± 7.00	34.44 ± 6.82	28.45 ± 5.75	**< 0.001**
	Coverage	0.76 ± 0.08	0.78 ± 0.09	0.75 ± 0.08	**0.048**
− 10	ACEA	25.69 ± 9.14	28.26 ± 9.83	22.73 ± 7.29	**< 0.001**
	LCEA	33.11 ± 6.94	35.90 ± 6.73	29.89 ± 5.71	**< 0.001**
	Coverage	0.79 ± 0.08	0.81 ± 0.08	0.78 ± 0.07	**0.020**
− 5	ACEA	29.74 ± 9.16	32.42 ± 9.81	26.65 ± 7.27	**< 0.001**
	LCEA	33.89 ± 6.77	36.66 ± 6.52	30.70 ± 5.57	**< 0.001**
	Coverage	0.81 ± 0.07	0.83 ± 0.07	0.80 ± 0.07	**0.016**
0	ACEA	33.84 ± 9.29	36.75 ± 9.83	30.48 ± 7.36	**< 0.001**
	LCEA	34.74 ± 6.65	37.41 ± 6.50	31.67 ± 5.40	**< 0.001**
	Coverage	0.84 ± 0.07	0.85 ± 0.07	0.82 ± 0.06	**0.02**
5	ACEA	37.46 ± 9.32	40.42 ± 9.80	34.05 ± 7.45	**< 0.001**
	LCEA	34.95 ± 6.55	37.42 ± 6.42	32.11 ± 5.48	**< 0.001**
	Coverage	0.84 ± 0.06	0.86 ± 0.06	0.83 ± 0.06	**0.004**
10	ACEA	41.15 ± 9.52	44.29 ± 9.86	37.55 ± 7.71	**< 0.001**
	LCEA	35.13 ± 6.61	37.47 ± 6.54	32.43 ± 5.62	**< 0.001**
	Coverage	0.85 ± 0.06	0.87 ± 0.07	0.84 ± 0.06	**0.008**
15	ACEA	44.68 ± 9.81	48.05 ± 10.06	40.80 ± 7.96	**< 0.001**
	LCEA	34.76 ± 6.67	37.00 ± 6.66	32.18 ± 5.71	**< 0.001**
	Coverage	0.84 ± 0.06	0.86 ± 0.06	0.84 ± 0.06	**0.023**
20	ACEA	48.16 ± 10.12	51.67 ± 10.30	44.12 ± 8.30	**< 0.001**
	LCEA	34.36 ± 6.89	36.51 ± 6.93	31.89 ± 6.00	**< 0.001**
	Coverage	0.84 ± 0.06	0.85 ± 0.07	0.83 ± 0.06	**0.033**
25	ACEA	51.56 ± 10.61	55.31 ± 10.81	47.24 ± 8.60	**< 0.001**
	LCEA	33.70 ± 7.04	35.77 ± 7.14	31.30 ± 6.14	** < 0.001**
	Coverage	0.84 ± 0.06	0.85 ± 0.07	0.83 ± 0.06	0.093
30	ACEA	54.91 ± 11.05	58.77 ± 11.20	50.46 ± 9.08	**< 0.001**
	LCEA	32.96 ± 7.14	34.81 ± 7.44	30.84 ± 6.19	**0.001**
	Coverage	0.83 ± 0.07	0.84 ± 0.07	0.82 ± 0.06	0.156

All data are shown as mean ± standard deviation

Bold: *p* < 0.05

*APP* anterior pelvic plane, *ACEA* anterior center-edge angle, *LCEA* lateral center-edge angle

**Fig. 2:**
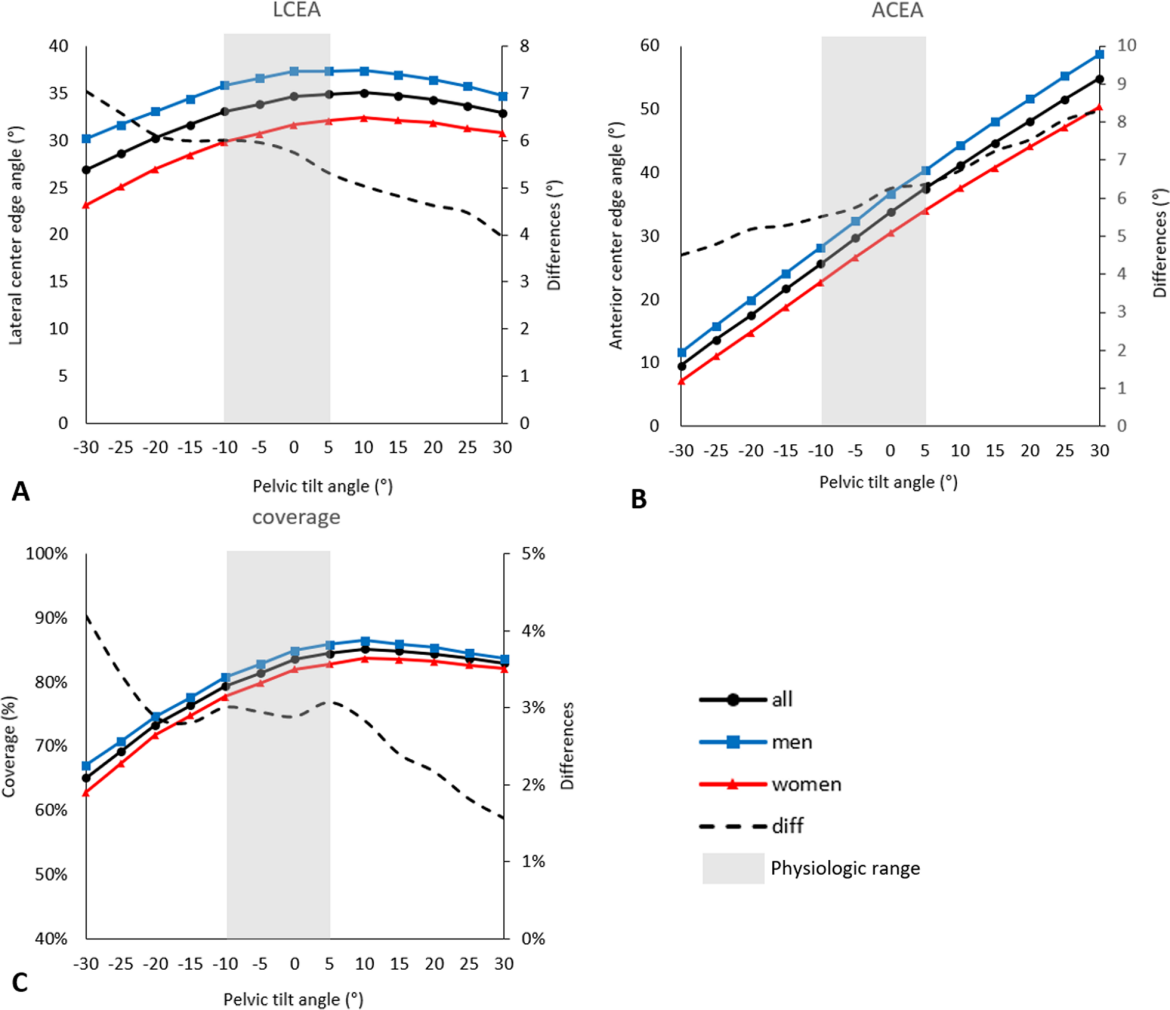
**A**–**C** The figures show changes in acetabular measurements according to APP tilt angle. Results of **A** lateral center-edge angle (LCEA), **B** anterior center-edge angle (ACEA), and **C** acetabular coverage

LCEA, ACEA, and acetabular coverage differed according to the APP tilt angle. LCEA and acetabular coverage showed maximum values at 10° which decreased as the pelvis was tilted anteriorly and posteriorly. ACEA showed a tendency to increase by an average of 3.6° for every 5° increase in the APP tilt angle (Fig. 2A–C). Within the physiologic range of APP tilt angles (− 10° to + 5°), both LCEA and ACEA, as well as coverage, increased as the angle increased. Within this range, the differences between the maximum and minimum values were 11.77° for ACEA, 1.84° for LCEA, and 5% for coverage. Sex differences were observed as the APP tilt angle increased, with LCEA and coverage decreasing while ACEA showed an increasing trend.

Pearson’s correlation analysis showed that acetabular coverage in both men and women was positively correlated with CEAs at all APP tilt angles. Additionally, LCEA demonstrated strong and very strong associations across all APP tilting angles, whereas ACEA showed a moderate association at angles ≥ 15° in men and ≥ 30° in women (Table 2).

**Table 2:** Result of Pearson correlation analysis of CEAs with acetabular coverage

APP tilt angle (°)	Men				Women			
	ACEA		LCEA		ACEA		LCEA	
	*R*	*p* value	*R*	*p* value	*R*	*p* value	*R*	*p* value
− 30	0.812	< 0.001	0.833	< 0.001	0.876	< 0.001	0.805	< 0.001
− 25	0.799	< 0.001	0.867	< 0.001	0.850	< 0.001	0.782	< 0.001
− 20	0.754	< 0.001	0.826	< 0.001	0.744	< 0.001	0.668	< 0.001
− 15	0.810	< 0.001	0.842	< 0.001	0.800	< 0.001	0.720	< 0.001
− 10	0.842	< 0.001	0.799	< 0.001	0.809	< 0.001	0.763	< 0.001
− 5	0.840	< 0.001	0.847	< 0.001	0.823	< 0.001	0.752	< 0.001
0	0.798	< 0.001	0.864	< 0.001	0.814	< 0.001	0.781	< 0.001
+ 5	0.721	< 0.001	0.885	< 0.001	0.777	< 0.001	0.807	< 0.001
+ 10	0.601	< 0.001	0.842	< 0.001	0.721	< 0.001	0.783	< 0.001
+ 15	0.568	< 0.001	0.874	< 0.001	0.735	< 0.001	0.806	< 0.001
+ 20	0.534	< 0.001	0.861	< 0.001	0.693	< 0.001	0.804	< 0.001
+ 25	0.474	< 0.001	0.857	< 0.001	0.618	< 0.001	0.782	< 0.001
+ 30	0.441	< 0.001	0.832	< 0.001	0.536	< 0.001	0.702	< 0.001

*APP* anterior pelvic plane, *ACEA* anterior center-edge angle, *LCEA* lateral center-edge angle, *R* correlation coefficient

The intra- and inter-observer reliabilities were as follows: 0.991/0.969 for acetabular coverage, 0.996/0.988 for LCEA, and 0.997/0.976 for ACEA.

## Discussion

In this 3D simulational study, LCEA, ACEA, and acetabular coverage were all statistically larger in men than in women (with the exception of coverage ≥ 25°). LCEA showed a strong and very strong correlation with coverage and did not require consideration of pelvic tilting within the physiologic range, but measurement differences became considerable with pelvic retroversion. In the case of ACEA, there is a strong and very strong correlation with acetabular coverage, as long as there is not a pathological anterior tilt of the pelvis. However, when interpreting ACEA, it is important to always consider pelvic tilting.

The influence of pelvic tilting on LCEA has shown different results in previous studies. Henebry et al. reported an increase in LCEA within the range of pelvic tilting from − 15° to + 15° in a cadaveric study, with a difference of 18.7° between the maximum and minimum values [8]. However, Tannast et al. suggested that pelvic tilting does not significantly affect the diagnosis of LCEA within the range of − 24° to + 24° based on their cadaveric study, and therefore, pelvic tilting does not need to be considered [26]. The reason for the discrepancies between these studies may be due to differences in the definition of the pelvic neutral position. Henebry et al. defined neutral position as distance of 4 cm between the symphysis pubis and the sacrococcygeal joint, while Tannast et al. defined pelvic inclination of 60° as neutral position. Another reason could be that sex was not controlled for in these studies. According to our study, the measurement of LCEA can vary by 4° to 7° between sexes. Therefore, if the sex ratio of the cadavers used in different studies varies, the results may also show differences.

According to the literature using radiographs or CT, the average APP tilt angle shows a range of − 3.8° to + 3° in the supine position [18, 24], and standing position shows 5°–6° more posterior tilting compared to supine [11, 24]. Therefore, we set the range of the physiologic APP tilt angle as − 10° to + 5°. Within this range, there is only a small difference in the maximum and minimum LCEA values, which is 1.84° (1.52° for men and 2.22° for women). Therefore, there is no need to consider individual or positional pelvic tilting when measuring LCEA, unless the patient has a pathologically abnormal pelvic orientation. However, as the APP tilt angle decreases below the physiologic range, the LCEA decreases progressively. Thus, the LCEA in a pelvis that is retroverted due to spinal disease [1, 19] or postural changes [25] may differ significantly from the LCEA in the neutral position. Therefore, when interpreting a patient's hip radiographs, it is crucial to consider the patient's position and pelvic orientation. On the other hand, for ACEA, there is an average increase of 3.6° for every 5° increase in APP tilt angle throughout the entire range, so pelvic orientation should always be taken into consideration [20].

**Fig. 3:**
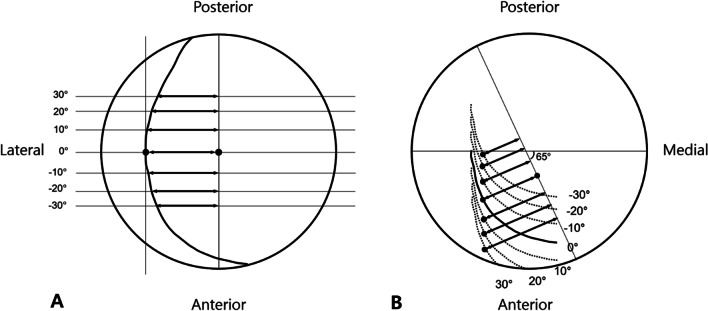
**A** and **B** Schematic composite showing changes of **A** lateral center-edge angle (LCEA) and **B** anterior center-edge angle (ACEA) according to anterior pelvic plane (APP) tilt angle

Although several studies have reported an association between LCEA on 2D simple radiographs and 3D femoral head coverage, they did not consider pelvic tilting [7, 22]. Moreover, although ACEA is associated with anterior acetabular coverage [21], to our knowledge, no studies have revealed its association with actual acetabular coverage. In our study, the LCEA and acetabular coverage showed a similar trend with a maximum value when the APP tilt angle was 10°, and decreased as the pelvis was tilted anteriorly or posteriorly. However, ACEA continued to increase even at an APP tilt angle of > 10°. This difference was due to the shape of acetabular coverage of the femoral head. Since LCEA is determined on the plane parallel to the APP passing through the most lateral acetabular rim, when the pelvis tilts anteriorly or posteriorly, it decreases along the shape of the laterally curvilinear acetabular rim (Fig. 3A). Changes in LCEA according to pelvic tilting showed a trend similar to the change in acetabular coverage of the gibbous moon shape, and LCEA showed strong and very strong associations (*R* > 0.6) with acetabular coverage at all APP tilt angles from − 30° to 30° (Table 2). However, since ACEA, which reflects the anterior coverage, is measured from the 65° side, the distance to the most protruded anterolateral point increased as the pelvic tilting increased (Fig. 3B). Therefore, ACEA differs from acetabular coverage and LCEA and shows a strong and very strong association with acetabular coverage at an APP tilting angle of 10° or less. Therefore, with the exclusion of ACEA in cases of excessive anterior tilting LCEA and ACEA are considered proper measurements to reflect actual acetabular coverage.

According to the existing literature, sex-based differences in CEA vary according to ethnicity. A study by Fisher et al. that used a relatively homogeneous white population showed women to have a larger LCEA [5], while a study from McWiliams et al. conducted in the UK showed LCEA to be greater in women without osteoarthritis [16]. Lavy et al. conducted a study with Africans and found no difference between the sexes [12], whereas in Asians, LCEA has clearly been found to be lower in women [9, 23, 29, 32]. In another study by Shi et al. that subdivided the Chinese population by age, there were generally more males than females (with statistically significant results for ages 19–40 and 51–60) [23]. Additionally, a study by Van Houcke et al. showed men to have higher CEAs in both Caucasian and Chinese populations [29], and another by Yoshimura et al. showed men to have higher CEAs than did women in Japan [32]. In Korea, men have larger LCEAs than women, and hip dysplasia is more common in women [9]. However, these studies had limitations in that they were evaluated solely using conventional X-rays. In our study of Koreans without hip joint disease, both the ACEA and LCEA were shown to be greater in men than in women, even when the APP tilt angle was corrected and compared. Considering that women are slightly more tilted anteriorly in the standing posture than men [4, 17], this result is consistent with the existing literature regarding Asians.

Our study had the following limitations. First, it was solely conducted among Koreans. Because racial differences in anatomy exist, the same analysis on other populations may show different results. Second, because this study targeted healthy people without hip disease, additional research is needed that includes patients with under- (hip dysplasia) or over- (pincer deformity) coverage. Third, this study did not account for situations such as the presence of fossa or position change of the hip center according to joint motion. There should be no outliers affecting the results when using only the normal acetabulum [10], but further validation is required. However, our study has the strength of being the first quantitative study to measure LCEA and ACEA values according to sex and APP tilt angle, and to elucidate their relationship with actual acetabular coverage. Another distinctive feature of our study, compared to previous cadaveric studies, is that we analyzed a range of − 30° to + 30° using 3D modeling, which is wider than the general physiologic range. Our study results can be helpful in predicting outcomes in cases of extreme pelvic tilting, such as severe kyphosis or Scheuermann's disease, or in cases where acetabular orientation is altered due to pelvic osteotomy.

## Conclusions

The LCEA and ACEA are adequate measurement methods that reflect actual acetabular coverage unless the pelvis is tilted excessively anteriorly. While pelvic tilting does not need to be considered for LCEA within the physiologic range, it should always be taken into account for ACEA, as it increases by an average of 3.6° for every 5° increase in APP tilt angle.

## Data Availability

The data sets used and/or analyzed in the current study are available from the corresponding author on reasonable request.

## Abbreviations

LCEA: Lateral center-edge angle of Wiberg
ACEA: Anterior center-edge angle, Lequesne’s acetabular index
2D: Two-dimensional
3D: Three-dimensional
APP: Anterior pelvic plane
